# Neural control of coronary artery blood flow by non‐adrenergic and non‐cholinergic mechanisms

**DOI:** 10.1113/EP090917

**Published:** 2023-04-08

**Authors:** Julia Shanks, Stian Thomson, Rohit Ramchandra

**Affiliations:** ^1^ Manaaki Manawa – The Centre for Heart Research, Department of Physiology University of Auckland Grafton Auckland New Zealand

**Keywords:** autonomic nervous system, coronary blood flow, non‐adrenergic, non‐cholinergic

## Abstract

Blood flow through the coronary vasculature is essential to maintain myocardial function. As the metabolic demand of the heart increases, so does blood flow through the coronary arteries in a dynamic and adaptive manner. Several mechanisms, including local metabolic factors, mechanical forces and autonomic neural control, regulate coronary artery blood flow. To date, neural control has predominantly focused on the classical neurotransmitters of noradrenaline and acetylcholine. However, autonomic nerves, sympathetic, parasympathetic and sensory, release a variety of neurotransmitters that can directly affect the coronary vasculature. Reduced or altered coronary blood flow and autonomic imbalance are hallmarks of most cardiovascular diseases. Understanding the role of autonomic non‐adrenergic, non‐cholinergic cotransmitters in coronary blood flow regulation is fundamental to furthering our understanding of this vital system and developing novel targeted therapies.

## Abstract (highlights)

- **What is the topic of this review?**
  How non‐adrenergic, non‐cholinergic neural mechanisms regulate coronary artery blood flow.
- **What advances does it highlight?**
  The main coronary arteries dynamically adapt to maintain adequate blood flow to the working myocardium. There is growing evidence for an important role of non‐classic neurotransmitters in regulating coronary blood flow. This review highlights current evidence for non‐adrenergic, non‐cholinergic control of coronary artery blood flow and our understanding of the dynamics of this system.

## 1 INTRODUCTION

The coronary circulation is essential for maintaining cardiac function. The viability of the myocardium and its ability to increase work relative to demand is directly linked to the capacity of the coronary circulation to increase blood flow. Coronary vascular resistance is continuously adapted to meet the metabolic demand of the myocardium. This regulation of coronary blood flow occurs through multiple mechanisms, including local metabolic factors, mechanical tissue forces, endothelial factors, circulating hormonal factors, and neural control. The importance of the coronary circulation has led to extensive study of these mechanisms in health and disease.

While metabolic and local endothelial factors play an important role in modulating coronary artery blood flow, this review will focus on the neural control of this critical organ. During states of increased metabolic demand, including physiological stresses such as exercise, and pathophysiological conditions, such as coronary artery disease, there is an essential role for neural control of the coronary circulation to maintain sufficient coronary blood flow. Cardiac ganglia are located at the origins of the coronary arteries and adjacent to the origin of the right, acute marginal coronary artery. Extensive innervation of all major branches of the autonomic nervous system, sympathetic, parasympathetic and sensory, are seen around the coronary vasculature. It is well known that autonomic nerves release many neurotransmitters at the end organ neuroeffector junction.

The majority of work on neural control of coronary flow has focused on the classic neurotransmitters noradrenaline (norepinephrine; NE) and acetylcholine (ACh). NE released from cardiac sympathetic nerves can either reduce coronary artery blood flow via α‐adrenergic receptor mediated vasoconstriction, or increase coronary flow by β‐adrenergic vasodilatation (Brandt et al., 2021; Di Carli et al., 1997; Heusch, 2011; Sun et al., 2002). A net NE vasoconstriction is thought to be predominant in the coronary vasculature at rest, but this may be different during periods of high sympathetic activity such as exercise, or in disease states. Parasympathetically released ACh dilates large coronary arteries via an M_3_‐muscarinic receptor nitric oxide pathway (Cox et al., 1983). However M_2_‐mediated coronary vasoconstriction has also been suggested in some species (Nasa et al., 1997). The role of NE and ACh in modulating coronary artery blood flow in health and disease has been extensively reviewed elsewhere (Baumgart & Heusch, 1995; Brandt et al., 2021).

**FIGURE 1:**
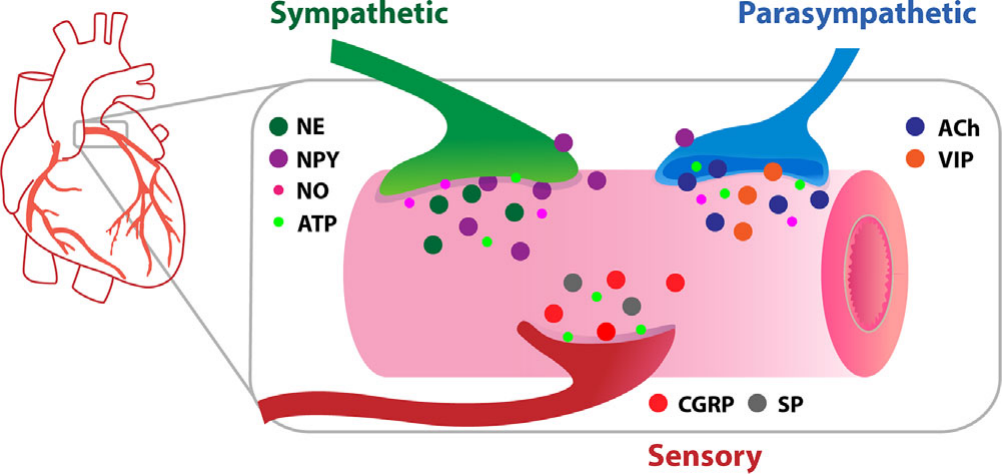
Schematic representation of the expanded model for neural control of coronary artery blood flow. ACh, acetylcholine; ATP, adenosine triphosphate; CGRP, calcitonin gene‐related peptide; NE, noradrenaline; NO, nitric oxide; NPY, neuropeptide Y; SP, substance P; VIP, vasoactive intestinal peptide.

Beyond the classic neurotransmitters there is growing evidence that part of the unique modulation of coronary flow may be due to the neural release of cotransmitters that allow fine modulation of coronary artery blood flow. In this context, we and others have shown that increases in coronary blood flow in response to direct stimulation or activation of various reflexes are not entirely NE or ACh mediated (Feliciano & Henning, 1998; Herring et al., 2019; Kennedy et al., 1997; Pachen et al., 2021; Pen et al., 2022; Seddon et al., 2009) indicating additional neuromodulators may be involved. This review will focus on neural control of coronary blood flow beyond classic neurotransmitters (Figure 1).

## 2 MEDIATORS RELEASED FROM THE SYMPATHETIC NERVES

Postganglionic sympathetic nerves travel with the coronary arteries in the epicardium before entering the myocardium. In postganglionic sympathetic nerves, neurotransmitter vesicles contain NE, adenosine triphosphate (ATP), nitric oxide (NO) and several polypeptides, including the vasoactive neuropeptide Y (NPY) (Burnstock, 2013).

NPY is released from sympathetic nerve terminals during high‐frequency stimulation (Kennedy et al., 1997). The NPY Y_1_ receptor on vascular smooth muscle cells promotes intracellular calcium release via the Gq–phospholipase C (PLC) pathway, causing vasoconstriction (Shigeri et al., 1995). NPY may also prevent endogenous vasodilators, including adenosine, adrenomedullin and NE, from acting via G_i_‐coupled β‐receptors (Prieto et al., 1997). This potential modulator of coronary blood flow has an important role in pathophysiology since higher NPY levels are associated with a higher level of microvascular resistance during myocardial infarction (MI). However, relative changes in NPY receptor activity or expression in the coronary vasculature post‐MI are unknown. Indeed, coronary sinus and peripheral venous NPY levels measured during primary percutaneous coronary intervention can act as a prognostic biomarker (Herring et al., 2019), highlighting the important role of NPY in pathophysiology.

In addition to direct coronary vascular effects, NPY can also act presynaptically on the Y_2_ receptor to inhibit NE release in a negative feedback mechanism (Toth et al., 1993). Moreover, Y_2_ receptors on parasympathetic nerves have been shown to mediate cross‐talk between the sympathetic and parasympathetic nervous systems. Y_2_ receptors on presynaptic parasympathetic neurons reduce ACh release via a protein kinase C (PKC)‐mediated mechanism (Shanks & Herring, 2013). Galanin, another cardiac sympathetic cotransmitter (Herring et al., 2012; Potter, 1987), may have presynaptic effects, but evidence for the direct effects of this neuromodulator on the coronary vasculature is lacking.

ATP co‐released with NE acts on postjunctional smooth muscle P2X_1_ receptors to evoke depolarisation. While the expression of other ATP receptors, including P2X_4_, P2X_5_ and P2X_7_, has been shown in other vascular smooth muscle beds, their role in vascular function and expression in the coronary arteries is unknown (Burnstock, 2017; Ralevic, 2012). However, a role for ATP signalling in the coronary artery beyond P2X_1_ cannot be ruled out. Elevation of circulating mRNA of the ATP P2X_7_ is closely associated with the severity of coronary artery stenosis, but its link to coronary artery flow is currently unknown (Shi et al., 2021). Synergistic cotransmission of NE and ATP potentiates a smooth muscle contractile response (Smith & Burnstock, 2004); however, synergistic actions of NE, ATP and NPY in the coronary vasculature are not yet known.

Adenosine, the breakdown product of ATP, has independent, vasodilatory actions (Hori & Kitakaze, 1991). The mechanism by which adenosine induces vasodilatation depends on species and vascular bed. In human coronary arterioles, adenosine shows an endothelium‐independent A_2_ receptor‐mediated dilatation. The interaction between A_1_, A_2a_ and A_2b_ receptors expressed on both vascular smooth muscle cells and endothelial cells is complex (Sato et al., 2005). A cardioprotective role for adenosine has been proposed for 40 years, with adenosine receptor agonists showing a protective role in rodent myocardial ischaemia (Lasley et al., 1990), and expression of the A_2a_ receptor is reduced in patients with coronary artery disease (Gaudry et al., 2020).

We know that these varied neurotransmitters are released from the sympathetic nerves, but the conditions under which they are released and their physiological roles remain under‐explored. Future studies are needed to understand the role non‐adrenergic neurotransmitters released from sympathetic nerves have on the dynamic modulation of myocardial blood flow.

## 3 MEDIATORS RELEASED FROM THE PARASYMPATHETIC NERVES

Parasympathetic nerves were traditionally thought to only innervate the sino‐atrial node and have a role in heart rate control. We now know that parasympathetic nerves extensively innervate the ventricles and coronary vasculature and release a variety of neurotransmitters (Coote, 2013). Research into non‐cholinergic parasympathetic control of coronary blood flow is predominately focused on vasoactive intestinal peptide (VIP). VIP‐immunoreactive neurons are expressed within parasympathetic perivascular nerve fibres, predominantly within the media–adventitia border of epicardial coronary arteries and other cardiac vasculature (Weihe et al., 1984). VIP is a highly potent vasodilator, 10 times more potent than ACh. In human and animal studies, intracoronary infusion of VIP resulted in vasodilatation within the arteries, increasing coronary blood flow (Popma et al., 1990). Emerging evidence suggests a vital role for VIP in maintaining coronary flow during periods of increased cardiac work.

VIP can be released from multiple sources, and direct release of VIP from vagal nerves has been shown. In anaesthetised canine experiments, vagal nerve stimulation (VNS) at high frequencies while under β‐adrenergic and muscarinic blockade increases coronary blood flow in the left circumflex artery (Feliciano & Henning, 1998), suggesting direct release from the nerves. The administration of a VIP antagonist attenuated this increase in flow. It is often suggested that the release of VIP occurs at higher stimulation frequencies (15–20 Hz), and VIP may be packaged in separate intracellular vesicles from those needed to release ACh, but direct evidence is lacking. While we know that VIP can be released during stimulation of the nerves, physiological initiators of VIP release from the vagus nerve are not well known. Reports indicate an increase in VIP release during myocardial infarction, potentially acting as a protective mechanism (Gyongyosi et al., 1997), and it is released into the coronary circulation in both normal and diseased hearts (Kupari et al., 2006), suggesting VIP may have a critical role in modulating cardiac function. In addition, VIP is also expressed in preganglionic sympathetic neurons (Mohney & Zigmond, 1998). While there are suggestions that it may be released from sympathetic neurons, there is no direct evidence for VIP release from postganglionc sympathetic fibres, to date.

Mechanistically, VIP‐induced vasodilatation in coronary vessels appears independent of the vascular endothelium, suggesting that, unlike ACh‐mediated vasodilatation, VIP does not act through an eNOS‐mediated mechanism (Luu et al., 1992). However, endothelial dependence varies by vascular bed and species (Henning & Sawmiller, 2001). The VIP receptors (VPAC1/2) are predominately G_s_ protein‐coupled receptors, likely stimulating adenylate cyclase and cAMP production to elicit vasodilatation in coronary arteries (Ganz et al., 1986). Changes in receptor expression on the coronary vasculature in cardiovascular disease are unknown. Similar to the sympathetic nerves, ATP is also co‐stored and co‐released with ACh and may act via P2X1 receptors to elicit smooth muscle contraction.

## 4 MEDIATORS RELEASED FROM THE SENSORY NERVES

In addition to the sympathetic and parasympathetic efferent nerves, afferent nerves are also present in the heart. Spinal projecting afferent nerve fibres have been identified as originating from coronary arteries and projecting to the dorsal root ganglia. The activation of sensory nerve endings in the coronary artery has been shown during mechanical stress, coronary artery occlusion and chemoreflex activation (Weaver et al., 1981). It is important to note that sensory nerve fibres can modulate coronary flow directly, beyond reflex control.

Calcitonin gene‐related peptide (CGRP) is a neuropeptide and potent vasodilator released from Aδ‐ and C‐fibre sensory nerve fibres (Gibbins et al., 1985; Kee et al., 2018). Immunostaining approaches have demonstrated the presence of CGRP in association with smooth muscles in the heart and coronary vasculature (Franco‐Cereceda et al., 1987). Exogenously applied CGRP increases coronary blood flow (Yaoita et al., 1994) via a direct action on the vasculature, independent of any systemic reflexes due to the changes in blood pressure or heart rate, as measured in humans by angiography (Ludman et al., 1991).

Substance P, a primary neurotransmitter of sensory afferent neurons, may also be a cotransmitter. Substance P is widely expressed in the peripheral nervous system, has been linked to multiple cardiovascular effects, and is thought to play a role in the control of coronary blood flow during ischaema–reperfusion and cardiovascular stress. Endogenous intracoronary infusion of substance P causes coronary vasodilatation in conscious humans (Weihe et al., 1984), and the potent vasodilator effects are likely linked to NO production in the endothelium (Seddon et al., 2009). In rats with cardiac sensory nerve denervation, and subjected to ischaemia–reperfusion, addition of substance P restored coronary artery flow (Ustinova et al., 1995). In both cases, while we know that CGRP and SP can be released from the afferent neurons, the physiological or pathological situations in which release occurs are not entirely known and need further investigation (Ustinova et al., 1995).

## 5 NEURONAL NITRIC OXIDE

Neuronal NO is a cotransmitter of both the sympathetic and parasympathetic nervous systems (Burnstock, 2013). The effects of NO on vascular tone are well established. However, the exact source of the NO and the upstream pathways involved in NO synthesis under different physiological and pathophysiological conditions are often overlooked or unknown. A large focus has been placed on the role of endothelial NO synthase (eNOS) in the regulation of coronary blood flow; however, more recent developments suggest an important role for neuronal NO synthase (nNOS) as a non‐adrenergic, non‐cholinergic (NANC) form of neural coronary control.

nNOS‐immunoreactive nerve fibres have been found within the coronary vasculature in animal models, but this has yet to be investigated in humans (Xavier, 2020), although nitrergic innervation has been observed in other human vascular beds. Coronary artery selective antagonism of nNOS reduced basal coronary artery blood flow in humans, indicating that the coronary vasculature may be under tonic nNOS control (Seddon et al., 2009). The effect of the nNOS‐antagonist was attenuated at higher cardiac workloads suggesting a more critical role as a mediator of basal flow (Khan et al., 2017). While there is currently little direct evidence for neuronal NO release in the coronary vasculature, nNOS knockout mice display an altered heart rate response to VNS, suggesting the presence of nitrergic fibres within the vagus nerve (Choate et al., 2001). More investigation is needed to show NO release from coronary perivascular nerves as cardiac nNOS has been identified in tissues other than nerves, including cardiac myocytes.

NANC neurotransmitters, including but not limited to VIP and substance P, likely signal, at least in part, by NO/eNOS pathways. nNOS and eNOS may have distinct roles in vascular function under different conditions. NO is currently utilised to treat several vascular conditions, including angina pectoris. Understanding the nuances of NO generation and its regulation by NANC neurotransmitters in different conditions could open new therapeutic pathways.

## 6 FUTURE DIRECTIONS

A mismatch between the demand of the working myocardium and the blood flow supply through the coronary arteries can significantly impact cardiovascular health. Reduced flow or reduced compliance of the coronary circulation is seen in most cardiovascular diseases, from coronary artery disease to heart failure, and hypertension (Ahmad et al., 2021; Heusch, 2022). Additionally, autonomic imbalance, characterised by increased sympathetic nerve activity and reduced parasympathetic nerve activity, is a primary feature and negative prognostic indicator of cardiovascular disease. The underlying causes of reduced flow through the coronary circulation in cardiovascular disease are complex, diverse and multifactorial, ranging from coronary atherosclerosis to endothelial dysfunction and altered neural hormonal signalling (Heusch, 2022). Understanding of the role NANC signalling and receptor expression have on coronary artery function in cardiovascular disease is limited and we need to better understand this. However, as our knowledge of autonomic neurotransmission increases, so does its apparent complexity. While NPY is predominantly a cotransmitter of the sympathetic nervous system, NPY also localises to interneurons and some parasympathetic neurons. ATP appears to be co‐stored in neurotransmitter vesicles of sympathetic, parasympathetic and sensory nerves. In addition to sensory fibres, substance P may also be expressed in parasympathetic fibres. Clearly, more work is needed to understand the complexity of this system better. Expanding our model to include the direct, synergistic and inhibitory actions of autonomic cotransmitters is necessary to further our understanding of this highly dynamic system. This expansion of knowledge has already generated a novel post‐MI prognostic indicator in circulating NPY levels, and the beneficial effects of VNS may go beyond the actions of ACh. Understanding the NANC regulation of the coronary circulation could further increase the potential for novel targeted therapies.

## AUTHOR CONTRIBUTIONS

All authors have read and approved the final version of this manuscript. All authors agree to be accountable for all aspects of the work in ensuring that questions related to the accuracy or integrity of any part of the work are appropriately investigated and resolved. All persons designated as authors qualify for authorship, and all those who qualify for authorship are listed.

## CONFLICT OF INTEREST

The authors declare no conflicts of interest.

## References

[eph13356-bib-0001] Ahmad, A. , Corban, M. T. , Toya, T. , Verbrugge, F. H. , Sara, J. D. , Lerman, L. O. , Borlaug, B. A. , & Lerman, A. (2021). Coronary microvascular dysfunction is associated with exertional haemodynamic abnormalities in patients with heart failure with preserved ejection fraction. European Journal of Heart Failure, 23(5), 765–772. 32949186 10.1002/ejhf.2010

[eph13356-bib-0002] Baumgart, D. , & Heusch, G. (1995). Neuronal control of coronary blood flow. Basic Research in Cardiology, 90(2), 142–159. 7646417 10.1007/BF00789444

[eph13356-bib-0003] Brandt, M. M. , Cheng, C. , Merkus, D. , Duncker, D. J. , & Sorop, O. (2021). Mechanobiology of microvascular function and structure in health and disease: Focus on the coronary circulation. Frontiers in Physiology, 12, 771960. 35002759 10.3389/fphys.2021.771960 PMC8733629

[eph13356-bib-0004] Burnstock, G. (2013). Chapter 3 ‐ Cotransmission in the autonomic nervous system. In R. M. Buijs , & D. F. Swaab (Eds.), Handbook of clinical neurology (Vol. 117, pp. 23–35). Elsevier. 10.1016/B978-0-444-53491-0.00003-1 24095113

[eph13356-bib-0005] Burnstock, G. (2017). Purinergic signaling in the cardiovascular system. Circulation Research, 120(1), 207–228. 28057794 10.1161/CIRCRESAHA.116.309726

[eph13356-bib-0006] Choate, J. K. , Danson, E. J. F. , Morris, J. F. , & Paterson, D. J. (2001). Peripheral vagal control of heart rate is impaired in neuronal NOS knockout mice. American Journal of Physiology. Heart and Circulatory Physiology, 281(6), H2310–H2317. 11709397 10.1152/ajpheart.2001.281.6.H2310

[eph13356-bib-0007] Coote, J. H. (2013). Myths and realities of the cardiac vagus. The Journal of Physiology, 591(17), 4073–4085. 23878363 10.1113/jphysiol.2013.257758 PMC3779103

[eph13356-bib-0008] Cox, D. A. , Hintze, T. H. , & Vatner, S. F. (1983). Effects of acetylcholine on large and small coronary arteries in conscious dogs. Journal of Pharmacology and Experimental Therapeutics, 225(3), 764–769. 6864532

[eph13356-bib-0009] Di Carli, M. F. , Tobes, M. C. , Mangner, T. , Levine, A. B. , Muzik, O. , Chakroborty, P. , & Levine, T. B. (1997). Effects of cardiac sympathetic innervation on coronary blood flow. New England Journal of Medicine, 336(17), 1208–1216. 9110908 10.1056/NEJM199704243361703

[eph13356-bib-0010] Feliciano, L. , & Henning, R. J. (1998). Vagal nerve stimulation releases vasoactive intestinal peptide which significantly increases coronary artery blood flow. Cardiovascular Research, 40(1), 45–55. 9876316 10.1016/s0008-6363(98)00122-9

[eph13356-bib-0011] Franco‐Cereceda, A. , Gennari, C. , Nami, R. , Agnusdei, D. , Pernow, J. , Lundberg, J. M. , & Fischer, J. A. (1987). Cardiovascular effects of calcitonin gene‐related peptides I and II in man. Circulation Research, 60(3), 393–397. 3495374 10.1161/01.res.60.3.393

[eph13356-bib-0012] Ganz, P. , Sandrock, A. W. , Landis, S. C. , Leopold, J. , Gimbrone, M. A. Jr. , & Alexander, R. W. (1986). Vasoactive intestinal peptide: vasodilatation and cyclic AMP generation. American Journal of Physiology, 250(5 Pt 2), H755–760. 3010741 10.1152/ajpheart.1986.250.5.H755

[eph13356-bib-0013] Gaudry, M. , Vairo, D. , Marlinge, M. , Gaubert, M. , Guiol, C. , Mottola, G. , Gariboldi, V. , Deharo, P. , Sadrin, S. , Maixent, J. M. , Fenouillet, E. , Ruf, J. , Guieu, R. , & Paganelli, F. (2020). Adenosine and its receptors: An expected tool for the diagnosis and treatment of coronary artery and ischemic heart diseases. International Journal of Molecular Sciences, 21(15), 5321. 32727116 10.3390/ijms21155321 PMC7432452

[eph13356-bib-0014] Gibbins, I. L. , Furness, J. B. , Costa, M. , MacIntyre, I. , Hillyard, C. J. , & Girgis, S. (1985). Co‐localization of calcitonin gene‐related peptide‐like immunoreactivity with substance P in cutaneous, vascular and visceral sensory neurons of guinea pigs. Neuroscience Letters, 57(2), 125–130. 2412189 10.1016/0304-3940(85)90050-3

[eph13356-bib-0015] Gyongyosi, M. , Kaszaki, J. , Nemeth, J. , Wolfard, A. , Mojzes, L. , & Farkas, A. (1997). Myocardial and gastrointestinal release of vasoactive intestinal peptide during experimental acute myocardial infarction. Coronary Artery Disease, 8(6), 335–342. 9347213 10.1097/00019501-199706000-00002

[eph13356-bib-0016] Henning, R. J. , & Sawmiller, D. R. (2001). Vasoactive intestinal peptide: Cardiovascular effects. Cardiovascular Research, 49(1), 27–37. 11121793 10.1016/s0008-6363(00)00229-7

[eph13356-bib-0017] Herring, N. , Cranley, J. , Lokale, M. N. , Li, D. , Shanks, J. , Alston, E. N. , Girard, B. M. , Carter, E. , Parsons, R. L. , Habecker, B. A. , & Paterson, D. J. (2012). The cardiac sympathetic co‐transmitter galanin reduces acetylcholine release and vagal bradycardia: implications for neural control of cardiac excitability. Journal of Molecular and Cellular Cardiology, 52(3), 667–676. 22172449 10.1016/j.yjmcc.2011.11.016 PMC3314977

[eph13356-bib-0018] Herring, N. , Tapoulal, N. , Kalla, M. , Ye, X. , Borysova, L. , Lee, R. , Dall'Armellina, E. , Stanley, C. , Ascione, R. , Lu, C.‐J. , Banning, A. P. , Choudhury, R. P. , Neubauer, S. , Dora, K. , Kharbanda, R. K. , Channon, K. M. , & Study OAMI . (2019). Neuropeptide‐Y causes coronary microvascular constriction and is associated with reduced ejection fraction following ST‐elevation myocardial infarction. European Heart Journal, 40(24), 1920–1929. 30859228 10.1093/eurheartj/ehz115 PMC6588241

[eph13356-bib-0019] Heusch, G. (2011). The paradox of alpha‐adrenergic coronary vasoconstriction revisited. Journal of Molecular and Cellular Cardiology, 51(1), 16–23. 21458461 10.1016/j.yjmcc.2011.03.007

[eph13356-bib-0020] Heusch, G. (2022). Coronary blood flow in heart failure: cause, consequence and bystander. Basic Research in Cardiology, 117(1), 1. 35024969 10.1007/s00395-022-00909-8 PMC8758654

[eph13356-bib-0021] Hori, M. , & Kitakaze, M. (1991). Adenosine, the heart, and coronary circulation. Hypertension, 18(5), 565–574. 1937658 10.1161/01.hyp.18.5.565

[eph13356-bib-0022] Kee, Z. , Kodji, X. , & Brain, S. D. (2018). The role of calcitonin gene related peptide (CGRP) in neurogenic vasodilation and its cardioprotective effects. Frontiers in Physiology, 9, 1249. 30283343 10.3389/fphys.2018.01249 PMC6156372

[eph13356-bib-0023] Kennedy, B. , Shen, G. H. , & Ziegler, M. G. (1997). Neuropeptide Y‐mediated pressor responses following High‐frequency stimulation of the rat sympathetic nervous system. Journal of Pharmacology and Experimental Therapeutics, 281(1), 291–296. 9103509

[eph13356-bib-0024] Khan, S. G. , Melikian, N. , Shabeeh, H. , Cabaco, A. R. , Martin, K. , Khan, F. , O'Gallagher, K. , Chowienczyk, P. J. , & Shah, A. M. (2017). The human coronary vasodilatory response to acute mental stress is mediated by neuronal nitric oxide synthase. American Journal of Physiology. Heart and Circulatory Physiology, 313(3), H578–H583. 28646032 10.1152/ajpheart.00745.2016 PMC5625168

[eph13356-bib-0025] Kupari, M. , Mikkola, T. S. , Turto, H. , Lommi, J. , & Ylikorkala, O. (2006). Vasoactive intestinal peptide—release from the heart and response in heart failure due to left ventricular pressure overload. European Journal of Heart Failure, 8(4), 361–365. 16310407 10.1016/j.ejheart.2005.10.008

[eph13356-bib-0026] Lasley, R. D. , Rhee, J. W. , Van Wylen, D. G. L. , & Mentzer, R. M. (1990). Adenosine A1 receptor mediated protection of the globally ischemic isolated rat heart. Journal of Molecular and Cellular Cardiology, 22(1), 39–47. 2325132 10.1016/0022-2828(90)90970-d

[eph13356-bib-0027] Ludman, P. F. , Maseri, A. , Clark, P. , & Davies, G. J. (1991). Effects of calcitonin gene‐related peptide on normal and atheromatous vessels and on resistance vessels in the coronary circulation in humans. Circulation, 84(5), 1993–2000. 1934374 10.1161/01.cir.84.5.1993

[eph13356-bib-0028] Luu, T. N. , Dashwood, M. R. , O'Neil, G. S. , Chester, A. H. , Muddle, J. R. , & Yacoub, M. H. (1992). Effect of vasoactive intestinal peptide and the distribution of receptors in human epicardial coronary arteries. Coronary Artery Disease, 3(3), 231–236.

[eph13356-bib-0029] Mohney, R. P. , & Zigmond, R. E. (1998). Vasoactive intestinal peptide enhances its own expression in sympathetic neurons after injury. Journal of Neuroscience, 18(14), 5285–5293. 9651211 10.1523/JNEUROSCI.18-14-05285.1998 PMC6793472

[eph13356-bib-0030] Nasa, Y. , Kume, H. , & Takeo, S. (1997). Acetylcholine‐induced vasoconstrictor response of coronary vessels in rats: A possible contribution of M2 muscarinic receptor activation. Heart and Vessels, 12(4), 179–191. 9559968 10.1007/BF02767046

[eph13356-bib-0031] Pachen, M. , Abukar, Y. , Shanks, J. , Lever, N. , & Ramchandra, R. (2021). Regulation of coronary blood flow by the carotid body chemoreceptors in ovine heart failure. Frontiers in Physiology, 12, 681135. 34122147 10.3389/fphys.2021.681135 PMC8195281

[eph13356-bib-0032] Pen, D. , Shanks, J. , Barrett, C. , Abukar, Y. , Paton, J. F. R. , & Ramchandra, R. (2022). Aortic body chemoreceptors regulate coronary blood flow in conscious control and hypertensive sheep. Hypertension, 79(6), 1275–1285. 35382553 10.1161/HYPERTENSIONAHA.121.18767

[eph13356-bib-0033] Popma, J. J. , Smitherman, T. C. , Bedotto, J. B. , Eichhorn, E. J. , Said, S. I. , & Dehmer, G. J. (1990). Direct coronary vasodilation induced by intracoronary vasoactive intestinal peptide. Journal of Cardiovascular Pharmacology, 16(6), 1000–1006. 1704974 10.1097/00005344-199012000-00021

[eph13356-bib-0034] Potter, E. (1987). Presynaptic inhibition of cardiac vagal postganglionic nerves by neuropeptide Y. Neuroscience Letters, 83(1‐2), 101–106. 3441289 10.1016/0304-3940(87)90223-0

[eph13356-bib-0035] Prieto, D. , Buus, C. , Mulvany, M. J. , & Nilsson, H. (1997). Interactions between neuropeptide Y and the adenylate cyclase pathway in rat mesenteric small arteries: role of membrane potential. The Journal of Physiology, 502(2), 281–292. 9263910 10.1111/j.1469-7793.1997.281bk.x PMC1159549

[eph13356-bib-0036] Ralevic, V. (2012). P2X receptors in the cardiovascular system. Wiley Interdisciplinary Reviews: Membrane Transport and Signaling, 1(5), 663–674.

[eph13356-bib-0037] Sato, A. , Terata, K. , Miura, H. , Toyama, K. , Loberiza, F. R. Jr. , Hatoum, O. A. , Saito, T. , Sakuma, I. , & Gutterman, D. D. (2005). Mechanism of vasodilation to adenosine in coronary arterioles from patients with heart disease. American Journal of Physiology. Heart and Circulatory Physiology, 288(4), H1633–H1640. 15772334 10.1152/ajpheart.00575.2004

[eph13356-bib-0038] Seddon, M. , Melikian, N. , Dworakowski, R. , Shabeeh, H. , Jiang, B. , Byrne, J. , Casadei, B. , Chowienczyk, P. , & Shah, A. M. (2009). Effects of neuronal nitric oxide synthase on human coronary artery diameter and blood flow in vivo. Circulation, 119(20), 2656–2662. 19433760 10.1161/CIRCULATIONAHA.108.822205

[eph13356-bib-0039] Shanks, J. , & Herring, N. (2013). Peripheral cardiac sympathetic hyperactivity in cardiovascular disease: Role of neuropeptides. American Journal of Physiology. Regulatory, Integrative and Comparative Physiology, 305(12), R1411–R1420. 24005254 10.1152/ajpregu.00118.2013 PMC3882692

[eph13356-bib-0040] Shi, X. X. , Zheng, K. C. , Shan, P. R. , Zhang, L. , Wu, S. J. , & Huang, Z. Q. (2021). Elevated circulating level of P2×7 receptor is related to severity of coronary artery stenosis and prognosis of acute myocardial infarction. Cardiology Journal, 28(3), 453–459. 32436587 10.5603/CJ.a2020.0074 PMC8169198

[eph13356-bib-0041] Shigeri, Y. , Nakajima, S. , & Fujimoto, M. (1995). Neuropeptide YY1 receptors‐mediated increase in intracellular Ca^2+^ concentration via phospholipase C‐dependent pathway in porcine aortic smooth muscle cells. Journal of Biochemistry, 118(3), 515–520. 8690710 10.1093/oxfordjournals.jbchem.a124938

[eph13356-bib-0042] Smith, N. C. E. , & Burnstock, G. (2004). Mechanisms underlying postjunctional synergism between responses of the vas deferens to noradrenaline and ATP. European Journal of Pharmacology, 498(1‐3), 241–248. 15364001 10.1016/j.ejphar.2004.07.055

[eph13356-bib-0043] Sun, D. , Huang, A. , Mital, S. , Kichuk, M. R. , Marboe, C. C. , Addonizio, L. J. , Michler, R. E. , Koller, A. , Hintze, T. H. , & Kaley, G. (2002). Norepinephrine elicits beta2‐receptor‐mediated dilation of isolated human coronary arterioles. Circulation, 106(5), 550–555. 12147535 10.1161/01.cir.0000023896.70583.9f

[eph13356-bib-0044] Toth, P. T. , Bindokas, V. P. , Bleakman, D. , Colmers, W. F. , & Miller, R. J. (1993). Mechanism of presynaptic inhibition by neuropeptide Y at sympathetic nerve terminals. Nature, 364(6438), 635–639. 8394510 10.1038/364635a0

[eph13356-bib-0045] Ustinova, E. E , Bergren, D. , & Schultz, H. D. (1995). Neuropeptide depletion impairs postischemic recovery of the isolated rat heart: Role of substance P. Cardiovascular Research, 30(1), 55–63. 7553724

[eph13356-bib-0046] Weaver, L. C. , Danos, L. M. , Oehl, R. S. , & Meckler, R. L. (1981). Contrasting reflex influences of cardiac afferent nerves during coronary occlusion. American Journal of Physiology, 240(4), H620–629. 6261593 10.1152/ajpheart.1981.240.4.H620

[eph13356-bib-0047] Weihe, E. , Reinecke, M. , & Forssmann, W. G. (1984). Distribution of vasoactive intestinal polypeptide‐like immunoreactivity in the mammalian heart. Cell and Tissue Research, 236(3), 527–540. 6205761 10.1007/BF00217219

[eph13356-bib-0048] Xavier, F. E. (2020). Nitrergic perivascular innervation in health and diseases: Focus on vascular tone regulation. Acta Physiologica, 230(1), e13484. 32336027 10.1111/apha.13484

[eph13356-bib-0049] Yaoita, H. , Sato, E. , Kawaguchi, M. , Saito, T. , Maehara, K. , & Maruyama, Y. (1994). Nonadrenergic noncholinergic nerves regulate basal coronary flow via release of capsaicin‐sensitive neuropeptides in the rat heart. Circulation Research, 75(4), 780–788. 7522988 10.1161/01.res.75.4.780

